# Impact of Physical Activity on Reporting of Childhood Asthma Symptoms

**DOI:** 10.1007/s00408-017-0049-7

**Published:** 2017-09-15

**Authors:** Benedicta Nneoma Nnodum, Meredith C. McCormack, Nirupama Putcha, Seungyoung Hwang, Laura M. Paulin, Emily P. Brigham, Ashraf Fawzy, Karina Romero, Gregory B. Diette, Nadia N. Hansel

**Affiliations:** 1 Johns Hopkins University School of Medicine, Baltimore, MD USA; 2 Johns Hopkins University Bloomberg School of Public Health, Baltimore, MD USA

**Keywords:** Asthma, Respiratory symptoms, Physical activity, Children

## Abstract

This study aims to determine the impact of physical activity on asthma symptom reporting among children living in an inner city. Among 147 children aged 5–12 years with physician-diagnosed asthma, we assessed asthma symptoms using twice-daily diaries and physical activity using the physical activity questionnaire for children during three 8-day periods (baseline, 3 and 6 months). Linear, logistic, and quasi-poisson regression models were used to determine the association between physical activity and asthma symptoms; adjusting for age, sex, race, BMI, caregiver’s education, asthma severity, medication use, and season. A 1-unit increase in PAQ score was significantly associated with reporting more nocturnal symptoms [risk ratio (RR): 1.03; 95% CI 1.00–1.06], daytime symptoms (RR: 1.04; 95% CI 1.00–1.09), being bothered by asthma (RR: 1.05; 95% CI 1.00–1.09), and trouble breathing (RR: 1.05; 95% CI 1.00–1.10). Level of physical activity should be taken into account in clinical management of asthma and epidemiological studies of asthma symptom burden.

## Introduction

Several studies suggest that children with more severe asthma are less likely to participate in physical activity [1, 2]. However, it is also possible that physical activity will trigger bronchoconstriction and increased awareness of respiratory limitation. Cross-sectional studies suggest that children reporting greater physical activity have increased likelihood of reporting any wheeze in the last 12 months using the International Study of Asthma and Allergy in Childhood (ISAAC) questionnaires [3–6]. These studies could not account for the effect of recent physical activity on concurrent asthma symptoms, or the impact of changing levels of physical activity on changes in reported asthma burden. It remains unclear whether higher levels of physical activity in children with asthma are associated with reporting more severe asthma symptoms. We used a longitudinal study design to determine the impact of physical activity levels on childhood asthma symptoms during three separate 1-week periods over 6 months among children ages 5–12 years. We hypothesized that when children with asthma are more physically active, they are more likely to report being bothered by their asthma symptoms.

## Methods

### Study Population

We recruited children aged 5–12 years living in Baltimore City with physician-diagnosed asthma who experienced asthma symptoms and/or required rescue medication use within the prior 6 months as part of Asthma-DIET study, a 6 months prospective cohort study assessing the role of indoor environmental exposures on asthma morbidity. Participants were assessed daily for three 8-day periods (baseline, 3 and 6 months), which ensures data collection for each participant during different seasons. Primary caregivers provided written informed consent and children provided assent prior to inclusion. This study was approved by the Johns Hopkins Institutional Review Board.

### Data Collection

Questionnaires were administered at baseline assessing demographics, medication use, and asthma severity based on National Asthma Education and Prevention Program (NAEPP) guidelines [7]. We measured height and weight and calculated body mass index (BMI, kg/m^2^). Spirometry was performed using the KoKo spirometer (nSpire Health, Inc., Longmont, CO) before and after albuterol administration. At least three acceptable and reproducible spirometry curves were obtained in accordance with American Thoracic Society guidelines [8]. Predicted values of forced expiratory volume (FEV_1_) and forced vital capacity (FVC) were calculated using the formulae of Hankinson et al. [9].

Asthma symptoms were obtained using the pediatric asthma diary (PAD), a validated daily questionnaire to assess asthma symptom burden [10], which each child completed with their parent/caregiver twice daily (daytime and nighttime) during each 8-day period. Research coordinators provided detailed instructions the first day of each time period and performed three home visits during the week to review the diaries and confirm proper completion. The daytime PAD questionnaire (Table 1) assessed frequency of breathing problems, activity limitation, bother of daytime symptoms on a 6-point Likert scale, absence from school, unscheduled doctor, emergency room or hospital visits, and the number of albuterol puffs used. The nighttime questionnaire assessed number of awakenings caused by asthma symptoms on a 4-point Likert scale and number of rescue inhaler puffs used.

**Table 1:** Daytime and nocturnal pediatric asthma diary (PAD) questionnaires

Daytime PAD questionnaire: asthma symptom scale
The following questions have a yes/no answer:
Were you absent from school any part of the day due to asthma?
Did you visit a doctor, emergency room or hospital for asthma (other than a scheduled visit to the doctor) or treated with prednisone during the previous 24 h?
The following questions are scored using the scale below:
How much of the time did you have trouble breathing today?
How much did your asthma bother you today?
How much of the time did your asthma limit your activity today (activities include any sort of physical activity: running, playing, jumping, sports, bike-riding, gym, etc.)?

Score	Symptom description
0	Symptoms none of the time, no bother and effect on daily activities at any time
1	Symptoms a little of the time, bothered me a little and activity limitation a little of the time
2	Symptoms some of the time, bothered me somewhat and activity limitation some of the time
3	Symptoms a good bit of the time, bothered me a good deal and activity limitation a good bit of the time
4	Symptoms most of the time, bothered me very much and activity limitation most of the time
5	Symptoms all of the time, bothered most of the time and effect on activities all of the time

Nighttime PAD questionnaire: nighttime awakenings
The following question is scored using the scale below:
Were you awoken by asthma (either during the night or in the morning)?

Score	Description
0	No awakenings
1	Once
2	More than once
3	Awake all night

Physical activity was assessed at the end of each 8-day monitoring period using the Physical Activity Questionnaire for Children (PAQ-C), a self-administered questionnaire that measures frequency of participation in different activities over 7 days that has shown high reliability and moderate validity in assessing frequency of physical activity in children [11]. The final PAQ composite score is the mean of 9 items each measured on a 5-point Likert scale.

### Statistical Analyses

Descriptive statistics were used to characterize the patient sample using proportions or means with standard deviations where appropriate. The primary outcomes were daytime and nocturnal asthma symptoms and frequency of albuterol inhaler used daily. The daytime and night time symptoms were scored between 0 (good control) and 5 (poor control) as in previous studies [10]. Nighttime asthma symptoms were defined by being woken up by asthma either during the night or in the morning (continuous Likert score). Frequency of rescue medication use was determined by combining the number of daytime and nighttime albuterol puffs (continuous daily puffs of albuterol). Secondary outcomes included the responses to the individual items of the questionnaire.

Log-transformed linear, logistic, and quasi-poisson regression models were used to determine the relationship between physical activity and reporting of daily asthma symptoms; with and without adjustment for age, gender, race, BMI, caregiver’s education, baseline asthma severity, inhaled corticosteroid use, and season. Interaction between physical activity and gender, BMI and baseline asthma severity were tested. Statistical significance was defined as *p* < 0.05. We conducted all analyses using SAS 9.4 (Cary, NC).

## Results

We analyzed data from 147 participants after excluding two children who had incomplete physical activity data (Table 2). Participants had a mean age of 9.5 years and were predominantly African-American (96%), and with moderate or severe persistent asthma (66%). Mean (SD) PAQ score at baseline was 2.7 (0.8), which was not significantly different at follow-up and reflects a moderate amount of physical activity. Males, younger children, those with lower BMI and more educated caregivers had a higher level of physical activity. Baseline asthma severity was not associated with level of physical activity. Inhaled corticosteroid use at baseline was not significantly associated with PAQ scores. Over the course of the study, participants reported awakening due to asthma on 7.3% of nights and bothered by their asthma symptoms on 17.9% of days.

**Table 2:** Baseline characteristics of children ages 5–12 years with asthma enrolled in the ASTHMA-DIET study

	*N* (%) or mean ± SD
Children’s characteristics	
Age	9.5 ± 2.3
Female	68 (46.3)
Race	
Black/African-American	141 (95.9)
Body mass index	20.1 ± 5.0
Underweight	8 (5.4)
Normal weight	69 (46.9)
Overweight	26 (17.7)
Obese	42 (28.6)
Asthma medication use	
Albuterol	96 (65.3)
Inhaled corticosteroid (ICS)	64 (43.5)
Other (cromolyn, leukotriene modifier, theophylline, oral corticosteroids)	41 (27.9)
Asthma severity^a^	
Mild intermittent	37 (25.2)
Mild persistent	13 (8.8)
Moderate persistent	50 (34.0)
Severe persistent	47 (32.0)
Health insurance	
Private insurance	12 (8.2)
Public insurance	133 (90.5)
Other	2 (1.4)
Lung function	
Pre-bronchodilator forced expiratory volume (FEV_1_), L	1.63 ± 0.49
Pre-bronchodilator forced vital capacity (FVC), L	1.98 ± 0.66
Pre-bronchodilator FEV_1_/FVC	0.84 ± 0.10
Caregiver’s characteristics	
Education	
Not high school graduate	42 (28.6)
High school graduate	72 (49.0)
At least some college	32 (21.8)
Household income (annual)	
< $25,000	59 (40.1)
$25,000–$50,000	19 (12.9)
> $50,000	3 (2.0)
Not reported	66 (44.9)

*SD* standard deviation

^a^Asthma severity based on symptoms, medication use, activity interference, and spirometry

In multivariable analysis adjusting for confounders, including baseline asthma severity, a 1-unit increase in PAQ score (increased physical activity) was significantly associated with reporting of more overall daytime asthma symptoms and more nocturnal symptoms (Table 3). Children with higher physical activity score were also significantly more likely to report being bothered by their asthma and have trouble breathing (Table 3). In univariate analysis, physical activity was associated with activity limitation due to asthma but this association no longer reached statistical significance after adjusting for confounders. We did not find an association between physical activity and likelihood of missing school, visiting the doctor for asthma, or frequency of rescue medication use. There was no evidence for effect modification of PAQ on asthma symptom reporting by gender, BMI, or baseline asthma severity (all *p*-interaction > 0.05).

**Table 3:** Association between increased physical activity (1-unit increase in physical activity questionnaire score) and reporting asthma symptoms among 147 children enrolled in the ASTHMA-DIET study

Symptom	Unadjusted		Adjusted	
	OR/RR (95% CI)	*p*	OR/RR (95% CI)	*p*
Primary outcomes				
Daytime asthma symptom diary scale	1.05 (1.01–1.09)^b,^*	0.01	1.04 (1.00–1.09)^b,^*	0.04
Nocturnal asthma symptom diary scale	1.03 (1.01–1.06)^b,^*	0.004	1.03 (1.00–1.06)^b,^*	0.01
Daily puffs of albuterol inhaler used	1.24 (0.89–1.72)^b^	0.21	1.13 (0.81–1.58)^b^	0.47
Secondary outcomes				
Absent from school due to asthma	1.04 (0.56–1.92)^a^	0.91	0.97 (0.54–1.74)^a^	0.91
Doctor’s visit due to asthma	0.99 (0.50–1.97)^a^	0.98	1.09 (0.58–2.02)^a^	0.79
Trouble breathing	1.05 (1.01–1.10)^b,^*	0.01	1.05 (1.00–1.10)^b,^*	0.02
Bother due to asthma	1.05 (1.01–1.09)^b,^*	0.02	1.04 (1.00–1.09)^b,^*	0.04
Activity limitation due to asthma	1.05 (1.01–1.09)^b,^*	0.02	1.04 (1.00–1.09)^b^	0.06

Adjusted models include terms for baseline age, gender, race, baseline BMI, caregiver’s education, baseline asthma severity, inhaled corticosteroid use, and season

*OR* odds ratio, *RR* risk ratio or relative risk, *CI* confidence interval

* p < 0.05

^a^Odds ratio

^b^Relative risk

## Discussion

In our study of inner city children, we found that participants with more severe asthma had a similar level of physical activity compared to those with mild disease, suggesting that in our cohort, asthma severity was not a major limiting factor to physical activity. However, in longitudinal follow-up, during periods when children were more physically active, they reported more daytime and nocturnal asthma symptoms, including more likely to be bothered by their asthma symptoms and more nighttime awakenings. In addition, future epidemiological studies of asthma should consider whether level of physical activity is a contributing factor or potential confounder in evaluation of factors linked to asthma symptom burden.

Children living in urban environments experienced a higher burden of asthma morbidity, and studies of human activity patterns show that the built environment can significantly affect an individual’s activity level [12]. In our study, children reported moderate physical activity and physical activity level did not vary by asthma disease severity. This finding supports a study carried out in an asthma camp in rural Nova Scotia, Canada which showed that physical activity did not differ between children (8–11 year old) with varying asthma severity [14].

While several studies have investigated the link between physical activity and asthma diagnosis [1–6, 15], few have studied the effect of physical activity on respiratory symptoms among children with asthma. Nystad et al. reported that physically active children aged 7–16 years with asthma were more likely to report having wheezed in the last 12 months [13]. Similarly, Mitchell et al. found that vigorous physical activity was associated with increased likelihood of reporting wheeze in the last 12 months in adolescents (aged 13–14 years) but not in younger children (aged 6–7 years) [3]. However, these studies did not address whether more recent physical activity is associated with reporting more asthma burden or rescue medication use. In addition, these studies did not address how differing degrees of physical activity may affect reporting of asthma symptoms. Our longitudinal study allowed assessment of the impact of changing levels of physical activity on changes in reported asthma symptoms, which adds evidence to the investigation of a causal relationship between physical activity and asthma burden. When the children were more active, they had both increased daytime symptoms including more trouble breathing and increased activity limitation. They were also more likely to have nighttime awakenings due to asthma, suggesting that the increased asthma burden was not only at the time of physical activity but continued through the night. Notably, we found no association between higher physical activity and increased absence from school or more doctor’s visits, suggesting that physical activity was associated with being bothered by asthma symptoms but not increased health care utilization. Furthermore, our results should not be interpreted to discourage physical activity in children with asthma, as a systematic review demonstrated that physical training programs were well tolerated and improved cardiopulmonary fitness and quality of life in people with stable asthma [18].

Our study had some limitations. While the PAQ has demonstrated high reliability and moderate validity as a self-report instrument [11], physical activity measured using an accelerometer is more reliable and accurate [16, 17]. Our study sample was comprised of mostly low-income inner city African-American children which limits generalizability of our findings to other socio-economic groups.

In conclusion, physically active children with asthma were more likely to report daytime asthma symptoms, nocturnal awakenings due to asthma, and being bothered by asthma symptoms, but were not more likely to miss school or report doctor visits for asthma. Physical activity should be ascertained and accounted for in clinical practice and epidemiological studies of asthma symptom burden.
